# Emerging roles of RNA:DNA hybrid regulation by mammalian ribonuclease H2 in replication stress and cancer

**DOI:** 10.1242/jcs.264148

**Published:** 2025-12-10

**Authors:** Rosanna J. Wilkins, Eva Petermann

**Affiliations:** ^1^ Department of Cancer and Genomic Sciences, School of Medical Sciences, College of Medicine and Health, University of Birmingham, Birmingham B15 2TT, UK; ^2^ Birmingham Centre for Genome Biology, University of Birmingham, Birmingham B15 2TT, UK

**Keywords:** DNA replication, R-loops, Genomic instability, DNA damage, Transcription–replication conflicts, RNase H2

## Abstract

Replication stress involves the slowing or stalling of the replication fork as DNA is copied during S phase. This stress can drive genomic instability, a cancer hallmark. RNA:DNA hybrids, such as R-loops and single genome-embedded ribonucleotides, are significant sources of replication stress. RNA:DNA hybrid homeostasis must therefore be tightly regulated through prevention and removal. Ribonuclease H2 (RNase H2) functions both in R-loop removal and excision of single ribonucleotides from genomic DNA. Recent research has generated new mechanistic insights into the functions of RNase H2 in the replication stress response, and implicated both loss and overexpression of RNase H2 in cancer development and therapy response. These findings help generate new models but also raise new questions. This Review explores the contribution of RNA:DNA hybrids to replication stress, the involvement of RNase H2 in regulating these structures, and the emerging roles of RNase H2 in replication stress response and cancer.

## Teaser

**Summary:** Control of RNA:DNA hybrids by RNase H2 helps protect genome stability. Recent insights into RNase H2 molecular functions and alterations in cancer shed new light on mechanisms of human disease.

## Introduction

DNA is duplicated to generate two identical copies via the process of eukaryotic DNA replication, after which the two copies are separated during mitosis and cell division. Replication forks copy DNA in a semi-discontinuous fashion, using CMG helicase to unwind DNA, while DNA polymerases ε and δ perform DNA synthesis (Lujan et al., 2016). The perturbation of DNA replication is known as replication stress (RS). RS comprises replication fork slowing, speeding or stalling, which can interfere with the completion of DNA synthesis and drive genomic instability (GIN), a cancer hallmark (Hanahan and Weinberg, 2011). RNA:DNA hybrids, where RNA hybridises with complementary template DNA, contribute significantly to RS, as they can interfere with replication fork progression or promote replication-associated DNA damage (reviewed in García-Muse and Aguilera, 2019; Petermann et al., 2022). RNA:DNA hybrid homeostasis must therefore be tightly regulated through their prevention and removal (García-Muse and Aguilera, 2019), as dysregulation is implicated in tumorigenesis, cancer progression and treatment resistance (Boros-Oláh et al., 2019).

One class of enzymes that regulates RNA:DNA hybrids is the ribonuclease H (RNase H) family – comprising RNase H1 and RNase H2 – which degrades the RNA moiety of an RNA:DNA hybrid (Cerritelli and Crouch, 2009). RNase H2 contributes the majority of nuclear RNase H activity (Arudchandran et al., 2000; Frank et al., 1998) and thus will be the focus of this Review, which explores the causes of RS, the role of RNA:DNA hybrids and recent research implicating RNase H2 in the RS response. We also explore the implications of both overexpression and loss of RNase H2 in cancer development and how this can influence responses to cancer therapies.

## Replication stress

RS sources include unrepaired DNA lesions, such as those caused by UV light or reactive oxygen species (ROS) (Collins, 1999; Gaillard et al., 2015). RS can occur after depletion of essential replication components like nucleotides or replication protein A (RPA), after deregulation of replication initiation, or following treatment with ribonucleotide reductase inhibitors such as hydroxyurea (HU) (Zeman and Cimprich, 2014; Kotsantis et al., 2018; Koç et al., 2004). Single-strand DNA breaks pose a significant threat during DNA replication, as they can lead to one-ended double-strand breaks (DSBs) when encountered by ongoing replication forks (Arnaudeau et al., 2001). Other RS sources include compacted chromatin and DNA secondary structures (Saxena and Zou, 2022; Williams et al., 2023). Transcription machinery, or transcriptional by-products such as R-loops or positive DNA supercoiling, can also hinder replication fork progression, and such events are categorised under the umbrella term transcription–replication conflicts (TRCs) (García-Muse and Aguilera, 2016).

Replication blocks can lead to replication fork collapse, with dissociation of the replication machinery and generation of cytotoxic DSBs (reviewed in Cortez, 2015). Other consequences of RS can range from single base pair mutations to aneuploidy (see Saxena and Zou, 2022 for a recent review). Mitotic entry in the presence of under-replicated DNA can cause improper sister chromatid segregation, mitotic aberrations and GIN (Saxena and Zou, 2022). RS can culminate in cell death or cellular senescence; however, this can be leveraged for cancer treatment, as it can restrict tumour proliferation (reviewed in Cybulla and Vindigni, 2023).

### Transcription–replication conflicts

Transcription must occur during S phase (Bertoli et al., 2013), because DNA replication requires the expression of genes such as histones (Nelson et al., 2002). Additionally, long genes can require a duration longer than one cell cycle to be transcribed (Helmrich et al., 2011). Therefore, some interference between replication and transcription is inevitable. Genomic regions that are repetitive, early replicating and highly transcribed are particularly vulnerable to TRCs (Helmrich et al., 2011). Transcriptional activity in S phase can promote transcription-associated recombination (Gottipati et al., 2008). Thus, transcription represents a potent source of endogenous RS, DNA damage and GIN, as well generating hotspots for recombination (García-Muse and Aguilera, 2016).

**Fig. 1.**
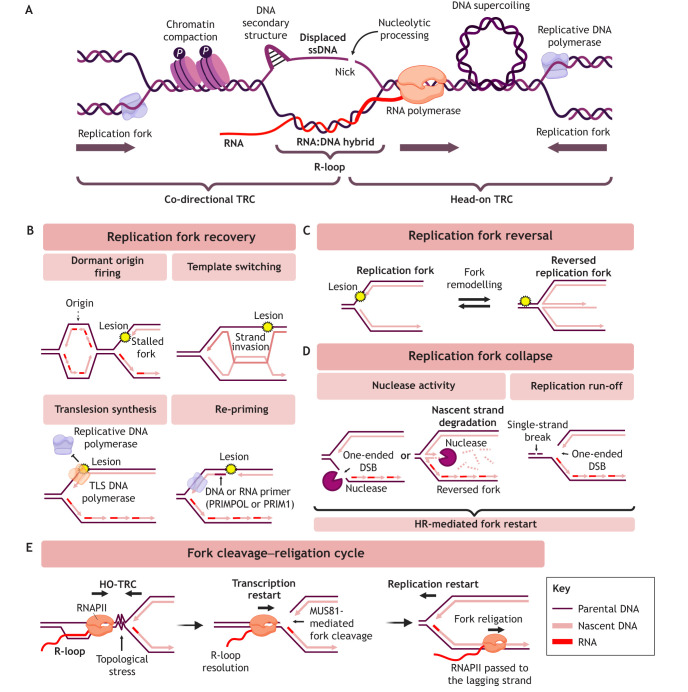
**Mechanisms of transcription–replication conflicts and recovery of stalled replication forks.** (A) TRCs occur in co-directional or head-on orientations. TRCs can result from direct collisions between replication and transcription machineries, R-loop-associated processes such as chromatin compaction (left), secondary structures or DNA breaks on the ssDNA of the R-loop (centre), or other transcription-associated processes such as positive DNA supercoiling (right). (B) Stalled forks can be passively rescued by local dormant origin firing or recovered through DNA damage tolerance mechanisms. Template switching is a recombination-based mechanism that involves copying the undamaged template through strand invasion. Translesion synthesis (TLS) DNA polymerases replicate across DNA lesions to recover the replication fork, and re-priming downstream of the lesion can facilitate restart. (C) Replication fork reversal by fork remodelling can stabilise stalled replication forks. (D) Persistent fork stalling can cause fork collapse, which commonly involves the generation of a one-ended DSB or nascent strand degradation by nucleases. Replication run-off at single-strand breaks can also generate a DSB. However, collapsed replication forks can be restarted using HR-mediated mechanisms. (E) A TRC-specific fork restart mechanism is the fork cleavage–religation cycle, where forks stalled by HO-TRCs associated with R-loops can restart following MUS81-generated cleavage of the replication fork. This can alleviate topological stress (left) and allow passage of the converged transcription machinery onto the lagging strand of the replication fork prior to religation and replication restart. Created in BioRender by Wilkins, R. (2025). https://BioRender.com/431i56t. This figure was sublicensed under CC-BY 4.0 terms.

The outcome of a TRC depends on its orientation (Fig. 1A). Head-on TRCs (HO-TRCs) arise when transcription and replication machineries travel towards each other, which canonically activates ataxia telangiectasia and Rad3-related (ATR) kinase signalling (Hamperl et al., 2017; Werner et al., 2025). If machineries travelling in the same direction collide, this causes co-directional TRCs (CD-TRCs), which predominantly activate ataxia telangiectasia mutated (ATM) kinase signalling (Hamperl et al., 2017). Eukaryotic transcription and replication machineries move at similar speeds, limiting CD-TRCs (Muniz et al., 2021). The frequency of HO-TRCs can be reduced by co-directional orientation of transcription and replication (Hamperl et al., 2017; Lang and Merrikh, 2021; Srivatsan et al., 2010). The incidence of HO-TRCs can be increased by transcriptional dysregulation, alterations in nucleotide metabolism (Andrs et al., 2023), dysregulation of origin firing (Hamperl et al., 2017) or dormant origin firing (Goehring et al., 2024). Despite the increased threat posed by HO-TRCs, CD-TRCs can also cause RS and GIN in particular contexts (Goehring et al., 2023; Merrikh et al., 2011).

TRCs can occur via direct mechanisms such as physical clashing of transcription machinery with the replisome. RNA polymerase (RNAP) removal from DNA allows cells to overcome TRCs (Poli et al., 2016; Pomerantz and O'Donnell, 2010), suggesting that the RNAP itself presents a major obstacle to fork progression. Transcription stress, where RNAP becomes stalled or backtracked, has been linked to replication-dependent DSBs (Dutta et al., 2011; Nudler, 2012). Indirect TRCs can occur through topological stress, R-loop formation or through the formation of DNA secondary structures associated with R-loops, like G-quadruplexes (Kumar et al., 2021). Topological stress in the form of positive supercoiling ahead of both processive machineries is suggested to be a major contributor to the damage caused by HO-TRCs (García-Muse and Aguilera, 2016), which can be reduced by DNA topoisomerase I (TOP1) activity (Tuduri et al., 2009) (Fig. 1A).

### Replication fork reversal, protection and restart

Cells have evolved mechanisms to stabilise, protect and recover blocked replication forks, which are important for the prevention of tumorigenesis (Fig. 1B,C). These mechanisms maintain forks in states that permit replication fork restart and enable time for DNA repair of replication-blocking lesions, or for the rescue of stalled replication forks by activating new origins of replication (Fig. 1B) (Ge et al., 2007). DNA lesions can be bypassed via DNA damage tolerance pathways to facilitate replication restart, including template switching to the undamaged sister chromatid, using a low-fidelity DNA polymerase for translesion synthesis, or re-priming to re-initiate DNA synthesis downstream of the blocking lesion (Fig. 1B) (Berti and Vindigni, 2016).

If lesions cannot be bypassed, stalled replication forks can be stabilised by replication fork reversal, whereby the three-way replication fork is remodelled into a four-way ‘chicken foot’ structure by DNA translocases and RAD51 recombinase (Fig. 1C) (Vujanovic et al., 2017; Zellweger et al., 2015). Fork reversal avoids further copying of damaged DNA, or fork collapse through replication runoff or nucleolytic attack (Fig. 1D) (Ray Chaudhuri et al., 2012). It also reduces single-stranded DNA (ssDNA) accumulation and creates a substrate for excision repair (Neelsen and Lopes, 2015). However, the nascent arm of the reversed fork is vulnerable to resection by nucleases like MRE11 and requires fork protection factors, such as the homologous recombination (HR) proteins BRCA1 and BRCA2, which limit nascent strand degradation at stalled forks (Schlacher et al., 2011).

Fork restart can occur directly from reversed replication forks, or RecQ family helicases such as RECQ1 (also known as RECQL) can remodel reversed forks back into three-way junctions (Berti et al., 2013). In the absence of restart, fork inactivation by dissociation of the replisome machinery and cleavage by endonucleases can lead to fork collapse generating a one-ended DSB (Fig. 1D) (Hanada et al., 2007). While DSB formation can be cytotoxic, collapsed forks can be rescued by the HR repair pathway break-induced replication (BIR) (Liao et al., 2018).

In the case of TRCs, forks stalled by an R-loop-associated RNAP in a head-on orientation can restart after endonuclease-mediated fork breakage by a process called the fork cleavage–religation cycle (Fig. 1E) (Andrs et al., 2023; Chappidi et al., 2020; Rao et al., 2024). RECQ1 and RECQ5 (RECQL5) revert reversed forks to three-way junctions, facilitating cleavage of the fork by the structure-specific endonuclease MUS81. This alleviates the TRC-associated topological barrier and facilitates transcription restart and RNAP progression onto the lagging strand (Andrs et al., 2023; Chappidi et al., 2020). Resolution of the associated R-loop and any associated DNA secondary structures is required for transcription restart (Isik et al., 2024; Rao et al., 2024). The RNA:DNA helicase senataxin (SETX) and DEAD-box RNA helicase DDX17 act sequentially to unwind the RNA:DNA hybrid component and resolve the R-loop at a TRC (Rao et al., 2024). RAD52 re-anneals the two parental strands together and DNA ligase IV (LIG4) and XRCC4 religate the replication fork before DNA synthesis is restarted through the action of PRIMPOL and POLD3. If the R-loop is not resolved, the MUS81-cleaved replication fork is degraded by DNA2, which can cause GIN rather than fork restart (Rao et al., 2024). Therefore, the resolution of R-loops by resolvers such as RNase H2 is important for preventing TRCs, GIN and thereby cancer development.

## R-loops

**Fig. 2.**
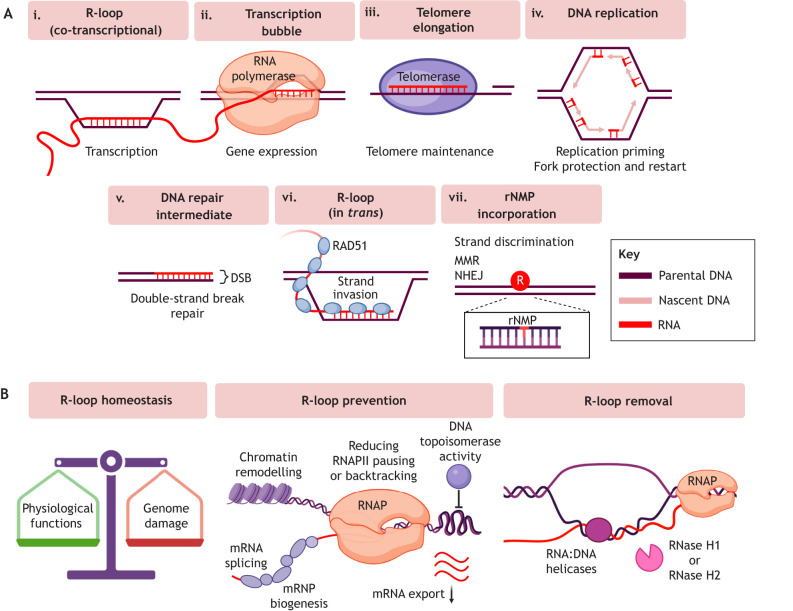
**Physiological functions of RNA:DNA hybrids and regulation of R-loops.** (A) Species of RNA:DNA hybrids in the genome include co-transcriptional R-loops (i). RNA:DNA hybrids also form within the transcription bubble of RNAP (ii) or within the active site of enzymes like telomerase (iii) during telomere end extension. Other species include RNA primers required during DNA replication (iv) or DNA repair intermediates (v). RNA:DNA hybrids are implicated in both major DSB repair pathways; for example, by protecting resected DNA ends. R-loops also form in *trans* (vi), mediated by RAD51. Single rNMPs are misincorporated at high frequencies in the genome (vii). rNMPs are implicated in strand discrimination, directing mismatch repair (MMR) and increasing ligation efficiency of NHEJ. (B) R-loop homeostasis is a fine balance between formation and removal (left), as R-loops have important physiological functions but are threats to genomic stability. R-loop prevention mechanisms such as chromatin remodelling, mRNA processing and export, promotion of RNAP processivity and DNA topoisomerase activity reduce the likelihood of nascent RNA re-hybridising with the DNA template (centre). R-loop resolution mechanisms (right) include RNA:DNA helicases, which can unwind RNA:DNA hybrids to resolve R-loops, and RNase H1 and RNase H2, which degrade the RNA component of the RNA:DNA hybrid. Created in BioRender by Wilkins, R. (2025). https://BioRender.com/qlies2t. This figure was sublicensed under CC-BY 4.0 terms.

R-loops in the genome can be a significant cause of TRCs and RS and could promote GIN in cancer. R-loop structures contain RNA:DNA hybrids that are several base pairs long (Fig. 2Ai). Other types of RNA:DNA hybrids are found within RNAP (Fig. 2Aii), where they stabilise and increase RNAP processivity during transcription (Nudler et al., 1997); within enzymes such as telomerase (Fig. 2Aiii); as RNA primers required to initiate or restart DNA synthesis (Fig. 2Aiv); or as intermediates formed during DNA repair (Fig. 2Av). RNA:cDNA hybrids can also form from reverse transcription in eukaryotic cells (Todd et al., 2020). R-loops are triple-stranded non-B DNA structures where nascent RNA re-anneals with template DNA to form the RNA:DNA hybrid while the non-template DNA strand is displaced as ssDNA, called a D-loop (reviewed in Petermann et al., 2022). Some techniques to map RNA:DNA hybrids, such as S9.6 anti-RNA:DNA hybrid antibody and RNase H1 hybrid binding domain-based tools, do not distinguish between R-loops and other species of longer RNA:DNA hybrid, and might be limited by non-specific binding to double-stranded RNA (Chédin et al., 2021). However, these mapping techniques can be complemented with approaches that detect the displaced ssDNA in the non-template strand of R-loops (Aguilera and García-Muse, 2012; Wu et al., 2022).

### R-loop formation

R-loops canonically occur co-transcriptionally behind transcription machinery, although anterior R-loops can form near backtracked RNAP (Zatreanu et al., 2019). R-loops typically extend for hundreds of base pairs – sometimes up to more than 2000 base pairs (Malig et al., 2020) – and require a free RNA 5′ end for their formation (Chen et al., 2017), therefore are considered distinct from the short RNA:DNA hybrid within the RNAP transcription bubble (Aguilera and García-Muse, 2012). In the ‘thread-back’ model, nascent RNA invades the complementary double-stranded DNA behind the RNAP (Aguilera and García-Muse, 2012). R-loops occupy ∼5% of the human genome and are profoundly dynamic, averaging half-lives of 10–20 min when formed co-transcriptionally (Crossley et al., 2020; Sanz et al., 2016). R-loops also form independently of transcription, in *trans* (Fig. 2Avi), when RNA invades a distal site of duplex DNA (Wahba et al., 2013). These transcription-independent R-loops are considered less stable and, thus, less threatening to genome stability (Kuzminov, 2018).

R-loop formation is further influenced by RNAP pausing and backtracking, DNA sequence, DNA modifications such as DNA methylation, DNA supercoiling, RNA processing and RNA editing (Sabino et al., 2022; Shiromoto et al., 2021). R-loop nucleation is facilitated by short (≥4) guanine clusters, and GC-rich regions promote R-loop stability (Roberts and Crothers, 1992), making transcription start and termination sites more susceptible to R-loop accumulation (Santos-Pereira and Aguilera, 2015; Sanz et al., 2016). Non-clustered G-rich DNA, especially in the non-template ssDNA strand of R-loops, promotes formation of highly stable G-quadruplex structures. In turn, G-quadruplexes can further stabilise R-loops (Duquette et al., 2004; Wanrooij et al., 2012). TRCs and their orientation can influence R-loop levels: HO-TRCs cause R-loop accumulation, whereas CD-TRCs lead to their resolution (Hamperl et al., 2017; Kumar et al., 2021). R-loop formation can also be initiated in a DNA sequence-independent manner at single-strand DNA breaks (Roy et al., 2010).

Transcriptional activity and the invasive potential of nascent RNA are increased in open chromatin, and mapping of mammalian genomes shows that R-loops are more consistently found in these regions (Sanz et al., 2016; Wahba et al., 2016). Genes transcribed by all three RNAPs – RNAPI, RNAPII and RNAPIII – can accumulate R-loops (Chan et al., 2014; El Hage et al., 2010), as can non-genic regions such as telomeres and centromeres (Feretzaki et al., 2020). R-loops also form in mitochondrial DNA (mtDNA), where their processing is suggested to regulate initiation of mtDNA replication (Posse et al., 2019).

### R-loops as a source of replication stress

R-loops can cause DNA damage by replication-dependent and -independent mechanisms. Cleavage of the exposed ssDNA strand of R-loops by structure-specific endonucleases such as xeroderma pigmentosum group F (XPF, also known as ERCC4), xeroderma pigmentosum group G (XPG, also known as ERCC5) and FEN1 (Cristini et al., 2019; Sollier et al., 2014), or by activation-induced cytidine deaminase (AID, also known as AICDA) (Su and Freudenreich, 2017), can cause DNA damage, GIN and accumulation of pro-inflammatory cytosolic nucleic acids in non-S phase and post-mitotic cells (Cristini et al., 2019, 2022; Crossley et al., 2023). However, most DNA damage caused by R-loops is replication dependent, arising predominantly from co-transcriptional R-loops, suggesting that R-loops pose the greatest risk at TRCs (Gan et al., 2011; Gómez-González and Aguilera, 2019). Indeed, transcription stress produced by R-loops can increase the propensity for TRCs (Shivji et al., 2018), and TRCs associated with R-loops are more threatening to genome stability than those without R-loop association (Hamperl et al., 2017). This suggests that R-loops are a major driver of TRC-mediated GIN.

What distinguishes a pathogenic from a non-pathogenic R-loop is currently unclear; however, R-loops induced by genetic or pharmacological perturbations, and R-loops that persist for longer, are in unusual locations or are of unusual length, have been proposed to be more detrimental (Chédin et al., 2021). In a reconstituted eukaryotic system, CMG helicase can bypass the RNA:DNA hybrid in both TRC orientations (Kumar et al., 2021). Leading-strand synthesis can continue over the displaced ssDNA of an R-loop, but presence of RNAP or a G-quadruplex on the displaced ssDNA increases the likelihood of replication fork stalling (Brickner et al., 2022). Thus, other features of R-loops – including G-quadruplexes, G-quadruplex-binding proteins, breaks in the displaced ssDNA strand or the ability of R-loops to anchor RNAP to the DNA – can contribute to impeding fork progression (Kumar et al., 2021; Zardoni et al., 2021). In addition, there is evidence that G-quadruplexes can persist even after RNA:DNA hybrid removal by RNase H1 in a subset of R-loops (Kumar et al., 2021), potentially leading to continued interference with DNA replication.

Chromatin status regulates R-loop homeostasis, and R-loop formation can reciprocally impact the chromatin landscape. R-loops have been linked to heterochromatin formation and are associated with phosphorylation of histone H3 at serine 10 (Castellano-Pozo et al., 2013; Nakama et al., 2012; Skourti-Stathaki et al., 2014). They might do this by influencing nucleosome positioning (Boque-Sastre et al., 2015), or by altering the recruitment of Polycomb repressive complexes 1 and 2 (Skourti-Stathaki et al., 2019) or the Tip60–p400 complex (Chen et al., 2015). Therefore, alterations in chromatin environments induced by R-loops, such as chromatin compaction, might also contribute to indirect TRCs (Castellano-Pozo et al., 2013; Skourti-Stathaki et al., 2014; Xu et al., 2021). Due to the risks of RS and GIN posed by R-loops, it is important that cells can regulate R-loop formation and R-loop resolution (Fig. 2B).

## Single genome-embedded ribonucleotides

Single ribonucleotides (ribonucleoside monophosphates, rNMPs), can become embedded in genomic DNA during DNA replication (Cerritelli and Crouch, 2016) (Fig. 2Avii), because DNA polymerase proofreading of rNMP incorporation is not absolute (Williams et al., 2012). Thus, rNMP incorporation is estimated at 10^6^ rNMPs per genome each cell cycle in mammalian cells, making misincorporated rNMPs the most common DNA lesion (Clausen et al., 2013a; Reijns et al., 2012). Multiple factors can influence rNMP misincorporation, such as the DNA polymerase, nucleotide pools and the DNA sequence (Balachander et al., 2020; Potenski and Klein, 2014). The rate of misincorporation can be altered by nucleotide pool imbalances, especially as the cellular ribonucleoside triphosphate concentration is 10–100 times higher than the deoxyribonucleoside triphosphate concentration (Nick McElhinny et al., 2010). In addition, different replicative polymerases have bias for incorporating specific rNMPs, as demonstrated by non-uniformity in the base of the rNMP misincorporated in different yeast strains (Balachander et al., 2020; Nick McElhinny et al., 2010; Potenski and Klein, 2014). For example, the rATP:dATP ratio is particularly high in *Saccharomyces cerevisiae*; however, rC and rG are preferentially misincorporated (Koh et al., 2015; Nick McElhinny et al., 2010).

Genome-embedded rNMPs can cause RS and GIN. At physiological pH, spontaneous phosphodiester bond hydrolysis is ∼100,000 times more likely in a ribonucleotide than a deoxyribonucleotide due to its reactive 2′-hydroxyl group (Li and Breaker, 1999), causing single-strand breaks and DSBs upon replisome collision (Schindler et al., 2023). The additional 2′-hydroxyl group of rNMPs can alter helix geometry due to steric crowding (Fu et al., 2019). Consequently, a single rNMP can block replicative polymerase progression in yeast (Clausen et al., 2013b; Watt et al., 2011). rNMPs can also cause transcription stress and have been associated with transcription-associated mutagenesis in yeast (Cho and Jinks-Robertson, 2017). rNMP incorporation greater than 5% within a specific region can furthermore prevent nucleosome formation (Hovatter and Martinson, 1987). As rNMPs can alter sequence recognition by DNA-binding proteins (Potenski and Klein, 2014), unrepaired rNMPs could theoretically alter the transcriptional landscape (Arana et al., 2012; Potenski and Klein, 2014). All these effects could contribute to RS and GIN. A threshold of genomic rNMPs tolerated during mouse development has been observed, where genomic rNMPs above this threshold lead to p53-dependent apoptosis and embryonic lethality. Below this threshold, increased rNMP levels trigger an innate immune response (Uehara et al., 2018). It is therefore important for cells to be able to remove rNMPs from the genome to safeguard genome stability and to prevent inflammation or cell death. This function is mainly performed by RNase H2.

## Ribonuclease H2

Cells have two specialised RNase H enzymes: RNase H1 and RNase H2 in eukaryotes, or RNase HI and RNase HII in prokaryotes (Kojima et al., 2018). Both enzymes are characterised by an RNase H fold in their catalytic domains and a highly conserved DEDD motif needed for phosphodiester bond cleavage in their active sites (Chapados et al., 2001; Ohtani et al., 1999). Eukaryotic RNase H1 is monomeric, whereas RNase H2 is heterotrimeric (Reijns et al., 2011; Shaban et al., 2010). RNase H1 has two distinct isoforms, which localise to either the mitochondria or the nucleus; the former is essential for mtDNA replication (Cerritelli et al., 2003). In contrast, RNase H2 is a nuclear enzyme, contributing to up to 80% of nuclear RNase H activity (Arudchandran et al., 2000; Frank et al., 1998). RNase H2, but not RNase H1, localises to sites of DNA replication (Bubeck et al., 2011; Nguyen et al., 2017). In eukaryotes, the RNASEH2A, RNASEH2B and RNASEH2C protein subunits of RNase H2 are encoded by *RNASEH2A*, *RNASEH2B* and *RNASEH2C* in mammals and by *RNH201*, *RNH202* and *RNH203* in yeast, respectively (Jeong et al., 2004; Reijns et al., 2011). In humans, biallelic hypomorphic variants of any one of the RNASEH2 genes causes the neuroinflammatory syndrome Aicardi–Goutières syndrome (AGS) (Crow et al., 2006).

RNASEH2A is the catalytic subunit. It contains the active site (Chapados et al., 2001) but is enzymatically inactive unless it is in complex with RNASEH2B and RNASEH2C (Chon et al., 2009; Jeong et al., 2004; Reijns et al., 2011). RNASEH2B and RNASEH2C are accessory subunits. They are less well conserved throughout species, and their exact functions remain to be completely elucidated (Chon et al., 2009). RNASEH2B is required for the nuclear localisation of RNASEH2A and RNASEH2C (Kind et al., 2014) and interacts with proliferating cell nuclear antigen (PCNA) via its non-canonical C-terminal PCNA-interacting protein-box (PIP-box) sequence. This localises the RNase H2 complex to replication foci without affecting RNase H2 catalytic activity (Bubeck et al., 2011). RNase H2 subunits interact in a stochiometric ratio and in a linear fashion, with RNASEH2C in the centre, acting as a platform for complex assembly (Chon et al., 2009). RNASEH2B and RNASEH2C can also form a stable heterodimer with unknown function (Chon et al., 2009). It has been speculated that upregulation of *RNASEH2A* can increase levels of active RNase H2 complex by providing additional RNASEH2A protein for binding the existing RNASEH2B–RNASEH2C heterodimer (Chon et al., 2009; Reijns et al., 2011).

### RNase H2 in R-loop regulation

R-loop homeostasis is tightly regulated in two ways: prevention and removal (Fig. 2B) (García-Muse and Aguilera, 2019). RNase H enzymes provide an important R-loop resolution mechanism (Cerritelli and Crouch, 2009). Loss of either RNase H1 or RNase H2 causes R-loop accumulation and GIN in yeast, but yeast RNase H2 can resolve R-loops on a global scale and has been described as the ‘housekeeping’ RNase H enzyme (Lockhart et al., 2019; Zimmer and Koshland, 2016). RNase H2 can also associate with RNAPII during transcription to remove co-transcriptional R-loops (Cristini et al., 2022).

Other factors can regulate R-loop formation, including those regulating RNAP pausing and backtracking, mRNA processing (Huertas and Aguilera, 2003; Li and Manley, 2005) and nuclear export of mature mRNA, such as via the transcription export (TREX) complex (Domínguez-Sánchez et al., 2011). mRNA processing factors can mitigate R-loop formation by binding and coating the nascent RNA, forming messenger ribonucleoproteins (mRNPs) (Salas-Armenteros et al., 2017). TOP1 and TOP2 play key roles in relieving torsional stress to prevent unscheduled R-loops (Tuduri et al., 2009). DNA repair factors such as BRCA2 and FANCD2 promote R-loop prevention and resolution by interacting with the TREX-2 mRNA export complex (Bhatia et al., 2014) or regulating the SRSF1 splicing factor (Olazabal-Herrero et al., 2020).

### Regulation of single genome-embedded ribonucleotides by RNase H2

RNase H1 requires four or more consecutive ribonucleotides in an RNA:DNA hybrid for its activity (Cerritelli and Crouch, 2009; Ohtani et al., 1999), whereas RNase H2 can also incise the phosphodiester backbone at the 5′ end of a single ribonucleotide in duplex DNA (Williams et al., 2016). Thus, initiation of ribonucleotide excision repair (RER), which excises single genome-embedded ribonucleotides from genomic DNA, is specific to RNase H2. A conserved valine 143 within the RNASEH2A active site fine-tunes substrate specificity (Baba et al., 2020). The 2′-hydroxyl group of the rNMP can physically interact with a GRG motif and tyrosine in RNASEH2A, whereas the active site of RNASEH2A contains the 5′ phosphate (Rychlik et al., 2010).

**Fig. 3.**
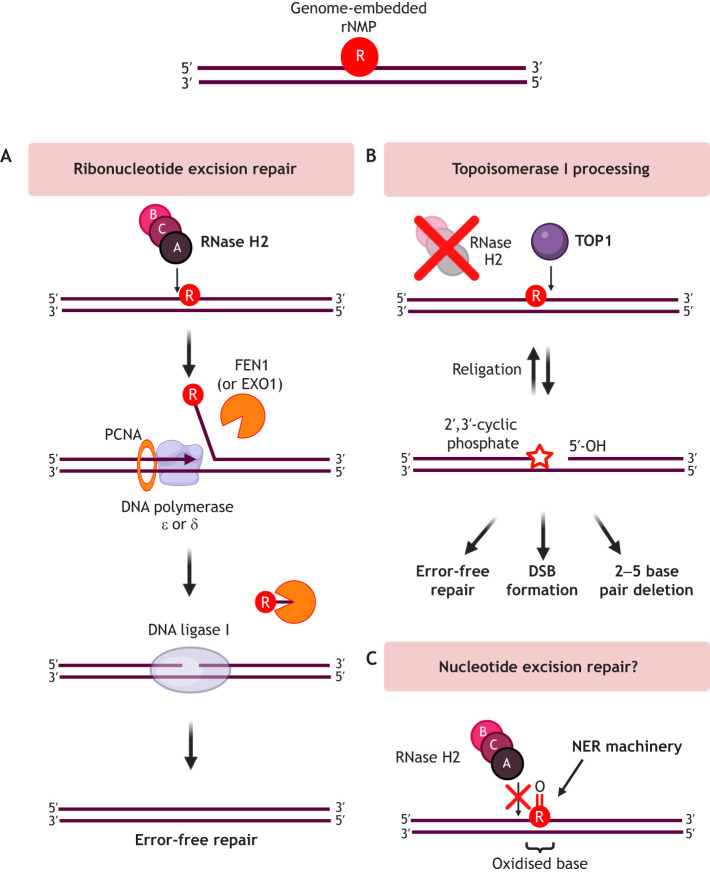
**Genome-embedded single ribonucleotide removal and repair.** (A) During RER, RNase H2 makes an incision 5′ of the rNMP (R, red). This is followed by strand displacement synthesis by a DNA polymerase, associated with PCNA. Flap endonuclease 1 (FEN1) or EXO1 cleaves the consequent flap structure, and the DNA nick is ligated by DNA ligase I, resulting in error-free repair of the rNMP. (B) If RNase H2 is lost, DNA topoisomerase I (TOP1) can reversibly cleave an rNMP at the 3′ end, generating DNA ends with a 2′,3′-cyclic phosphate (star) and 5′-hydroxyl (5′-OH) group. Downstream processing can sometimes lead to error-free repair, but this mechanism is associated with mutational signatures such as 2–5 base pair deletions, as well as DSBs, which require repair. (C) Human RNase H2 has difficulty processing oxidised rNMPs. At these lesions, NER factors have been implicated in removing rNMPs *in vitro*, although the prevalence of NER in ribonucleotide excision in human cells remains under debate. Created in BioRender by Wilkins, R. (2025). https://BioRender.com/xgfps3f. This figure was sublicensed under CC-BY 4.0 terms.

RER is the predominant pathway of rNMP removal (Fig. 3A). First, RNase H2 incises the phosphodiester backbone 5′ of the rNMP, then DNA strand displacement synthesis occurs by DNA polymerase δ, generating a DNA flap. This structure is cleaved by FEN1, and the nick is ligated by DNA ligase I (LIG1). Alternatively, DNA polymerase ε and exonuclease 1 (EXO1) can replace DNA polymerase δ and FEN1, respectively, thus RER occurs on both the leading and lagging DNA strands (Sparks et al., 2012). Human RNA helicase DDX3X has recently been suggested to have RNase H2-like activity and initiate RER *in vitro* (Riva et al., 2020).

In the absence of RNase H2, TOP1-mediated cleavage 3′ of the rNMP initiates a backup removal pathway (Fig. 3B) (Kellner and Luke, 2020). The initial TOP1 nick is characterised by 2′,3′-cyclic phosphate and 5′-hydroxyl ends, which are susceptible to nucleolytic processing, often leading to ribonucleotide-dependent mutagenesis and GIN, and specific mutational signatures such as 2–5 base pair deletions (Clark et al., 2011; Kim et al., 2011; Nick McElhinny et al., 2010; Zimmermann et al., 2018). TOP1 processing of genome-embedded rNMPs can result in DSBs that require HR repair (Huang et al., 2017). Another backup pathway suggested for RER is nucleotide excision repair (NER) (Fig. 3C) (Cai et al., 2014; Sassa et al., 2019). This could prove a useful strategy in some contexts – for example, when an rNMP is oxidised, which human RNase H2 has difficulty processing (Sassa et al., 2019).

The RER function of RNase H2 has been proposed to prevent embryonic lethality, RS and tumorigenesis in mammals (Reijns et al., 2012; Uehara et al., 2018). In yeast, a separation-of-function mutant of RNASEH2A that is RER defective has been employed to investigate the contributions of both RNase H2 functions in RER and R-loop removal (Chon et al., 2013). This separation-of-function mutation has since been recreated in human and mouse cells; however, in human cells the RER-defective mutation can also slightly impair the R-loop-resolving activity (Benitez-Guijarro et al., 2018).

## Roles of RNase H2 in replication stress

Although RNase H2 clearly functions in DNA replication, there is no direct evidence of cell cycle regulation of mammalian RNase H2. However, RNase H2 protein levels are increased in cycling cells, compared to non-cycling cells, supporting the idea that RNase H2 is a DNA replication and repair enzyme (Marsili et al., 2021; Reijns et al., 2012).

### RNase H2 in resolving RNA:DNA hybrids at the replication fork

**Fig. 4.**
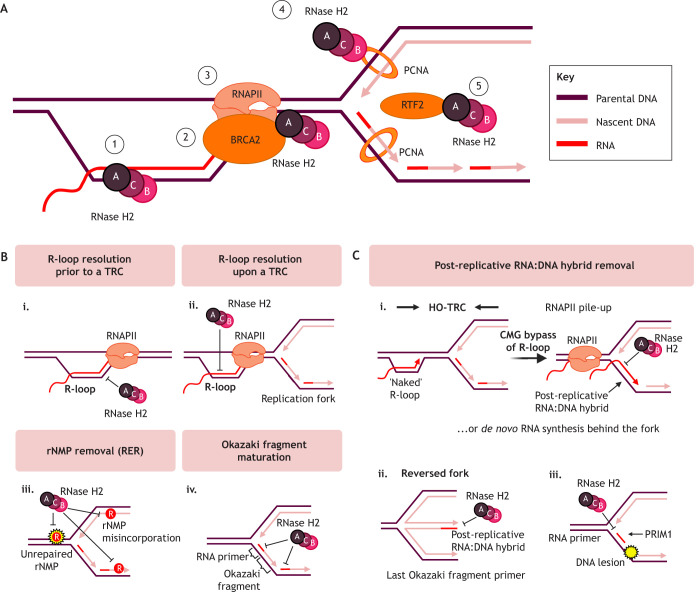
**RNase H2 localisation and potential functions at replication forks.** (A) Mechanisms of RNase H2 localisation to TRCs. Direct substrate interaction through the active site of RNASEH2A can recruit RNase H2 (1). Alternatively, RNase H2 can be recruited to transcription machinery through interactions with BRCA2 (2) or RNAPII (3), or to the replisome through direct interactions with PCNA via PIP-boxes in RNASEH2A or RNASEH2B (4), or through recruitment by RTF2 (5). (B) RNase H2 can resolve an R-loop prior to (i) or upon (ii) interference with DNA replication. RNase H2 can initiate RER to remove genomic rNMPs, either as they are being incorporated, or upon their interference with DNA replication (iii). RNase H2 is implicated in removing the RNA primer required for lagging strand DNA synthesis during Okazaki fragment maturation (iv). (C) RNase H2 has been implicated in removing post-replicative RNA:DNA hybrid species that can interfere with replication fork restart. These species can arise from CMG bypass of a naked R-loop or *de novo* RNA synthesis behind the fork (i). Upon fork reversal, the last unprocessed Okazaki fragment primer can form post-replicative RNA:DNA hybrids that interfere with fork restart (ii). Unrestrained RNase H2 has also been implicated in degrading RNA primers required for fork restart (iii). Created in BioRender by Wilkins, R. (2025). https://BioRender.com/55rko5g. This figure was sublicensed under CC-BY 4.0 terms.

RNase H2 recruitment to replication forks has been demonstrated by nascent chromatin capture (Alabert et al., 2014). RNase H2 can be recruited directly to the replication fork through substrate interactions with RNASEH2A (Fig. 4A). Other factors might regulate its localisation: for example, BRCA2 can recruit RNase H2 to DSBs (D'Alessandro et al., 2018), therefore could also localise RNase H2 to stalled replication forks. BRCA2 can interact with RNAPII, which could help recruit RNase H2 to the transcription machinery and TRCs (Cristini et al., 2022; Goehring et al., 2024; Shivji et al., 2018). In addition, the RNASEH2B–PCNA interaction can localise RNase H2 to replication forks and potentially to DNA repair sites where PCNA accumulates (Bubeck et al., 2011; Kind et al., 2014). This interaction is independent of RS, suggesting an rNMP surveillance mechanism during unperturbed replication (Bubeck et al., 2011). A PIP-box in RNASEH2A can also bind PCNA *in vitro* (Tian et al., 2024). Alternatively, replication termination factor 2 (RTF2), a replisome component, can recruit RNase H2 to replication forks independently of RS in mammalian cells (Conti et al., 2024).

The ability of RNase H2 to resolve RNA:DNA hybrids could prevent TRCs from occurring in the first place (Fig. 4Bi) or remove obstacles after fork stalling (Fig. 4Bii) (Audoynaud et al., 2023; Heuzé et al., 2023). Alternatively, RNase H2 could mitigate RS by excising single rNMPs from the genome (Fig. 4Biii). RNase H2 could also remove RNA:DNA hybrids during unperturbed replication, functioning in an Okazaki fragment maturation (OFM) backup pathway (Sun et al., 2023), and might be involved in promoting FEN1 flap cleavage activity during canonical OFM in human cells (Fig. 4Biv) (Tian et al., 2024).

RNA:DNA hybrids have been implicated in replication fork stalling in response to RS-inducing agents camptothecin (CPT, a topoisomerase I inhibitor) and HU (Kemiha et al., 2021). Post-replicative RNA:DNA hybrids behind stalled forks – which have been visualised using electron microscopy – could result from *de novo* transcription or from pre-existing RNA re-annealing to complementary ssDNA in transcriptionally active regions (Stoy et al., 2023). The same co-transcriptional R-loop that stalls a replication fork can be converted to a post-replicative RNA:DNA hybrid after bypass during a head-on encounter with slow-moving replisomes (Fig. 4Ci) (Heuzé et al., 2023).

RNase H2 has been suggested to promote fork progression or restart in CPT- or HU-treated cells by degrading post-replicative RNA:DNA hybrids originating from pre-existing transcripts or the Okazaki primer (Audoynaud et al., 2023; Goehring et al., 2024; Heuzé et al., 2023). Transcription inhibition restores controlled nascent strand resection in human cells depleted of RNase H2, suggesting that RNase H2 activity is required to alleviate co-transcriptional RNA:DNA hybrids that block fork recovery (Heuzé et al., 2023). While these mechanisms are transcription dependent, the last un-matured Okazaki primer, deposited during replication, can also be a source of post-replicative RNA:DNA hybrids (Fig. 4Cii), as demonstrated at stalled forks in fission yeast, where the hybrids help recruit protective factors at reversed forks but must be eventually removed by RNase H1 or RNase H2 to promote restart (Audoynaud et al., 2023). Thus, whether the RNA:DNA hybrids accumulate before or after the stalled fork, or both, is complex and likely context dependent. For example, HU-induced RNA:DNA hybrids are postulated to form in response to HO-TRCs (Andrs et al., 2023; Goehring et al., 2024). RNase H2 also accumulates at telomeres to counteract telomeric R-loop-associated RS (Graf et al., 2017). Regardless of the mechanism of RNA:DNA hybrid formation at stalled forks, their appropriate removal is required for fork recovery.

Persistent post-replicative RNA:DNA hybrids could interfere with fork recovery by preventing the controlled resection required for HR-mediated restart. Therefore, their removal at the appropriate time promotes restart (Andrs et al., 2023; Audoynaud et al., 2023; Chappidi et al., 2020; Heuzé et al., 2023). Unrestrained RNase H2 activity has also been implicated in degrading RNA primers required for re-priming (Fig. 4Ciii) (Conti et al., 2024). Other RNA:DNA hybrid resolvers, such as senataxin (Rao et al., 2024) and DDX17 (Boleslavska et al., 2022), have recently been linked to replication fork restart in yeast and human cells.

### RNase H2 in DSB repair

RNase H2 is implicated in both major DSB repair pathways – HR and non-homologous end joining (NHEJ) – as loss of RNase H2 causes resection defects at DSBs (Daley et al., 2020). In mammalian cells, BRCA2 recruits RNase H2 to DSBs to resolve RNA:DNA hybrids that can otherwise interfere with HR by preventing RAD51 nucleofilament formation (D'Alessandro et al., 2018). In contrast, activity of both RNase H enzymes has been implicated in inhibiting RNA-templated HR repair in yeast, by degrading transcript RNA that serves as an undamaged template (Keskin et al., 2014). It is estimated that 65% of DSBs repaired by NHEJ incorporate rNMPs, so that NHEJ can remain functional during times of stress where nucleotide pools are imbalanced (Pryor et al., 2018). RNase H2-mediated rNMP incision elevates ligation efficiency during NHEJ (Pryor et al., 2018). Thus, RNA:DNA hybrid removal by RNase H2 is implicated in maintaining genome stability both by preventing RS and by promoting DNA repair, processes that could be exploited for cancer therapy.

## Exploiting RNA:DNA hybrid homeostasis in cancer therapy

Unscheduled RNA:DNA hybrids and R-loops accumulate across a range of solid tumours and haematological malignancies (Boros-Oláh et al., 2019). RS and GIN caused by R-loops could drive mutagenesis and tumour development (Elsakrmy and Cui, 2023). Additionally, R-loop dysregulation could promote tumorigenesis by rewiring the transcriptional landscape (Wells et al., 2019). RNA:DNA hybrid and R-loop accumulation is promoted by oncogenes and proliferation factors (Kotsantis et al., 2016; Stork et al., 2016). The tumour suppressor p53 is speculated to be a negative regulator of R-loop levels, TRCs and R-loop-mediated GIN (Panatta et al., 2022; Yeo et al., 2016). For example, in human papillomavirus (HPV)-positive cervical cells, R-loop levels are increased by the HPV E6 oncoprotein, which suppresses p53 expression (Templeton and Laimins, 2023). Increased R-loop levels observed in castration-resistant prostate cancer (CRPC), as compared to localised prostate cancer, might link R-loop accumulation to prostate cancer progression (Kimura et al., 2022).

It has therefore been suggested that cancer cells rely on upregulation of proteins that help tolerate increased R-loop levels, maintaining DNA damage, RS and GIN at a sub-lethal level (Boros-Oláh et al., 2019; Elsakrmy and Cui, 2023; Kimura et al., 2022). In multiple myeloma, genes involved in resolution of R-loops and TRCs are overexpressed (Dutrieux et al., 2021), and hepatoma cells upregulate poly(ADP-ribose) polymerase (PARP) to modulate R-loop levels and confer resistance to CPT treatment (Ye et al., 2021). R-loop accumulation might be targetable in haematological malignancies that harbour mRNA splicing factor mutations associated with R-loop accumulation (Liu et al., 2024; Nguyen et al., 2017, 2018). R-loop accumulation activates ATR and PARP1, making ATR or PARP inhibition an attractive strategy in cells with mutations in spliceosome components such as U2 small nuclear RNA auxiliary factor 1 (U2AF1) or serine/arginine-rich splicing factor 2 (SRSF2) (Liu et al., 2024; Nguyen et al., 2017, 2018). A recent phase I/II clinical trial has investigated ATR inhibition in haematological malignancies stratified by splicing factor mutations (Brunner et al., 2021).

Exacerbating R-loop-associated TRCs by stabilising G-quadruplexes with ligands such as pyridostatin has been therapeutically explored in multiple myeloma (Dutrieux et al., 2025). High R-loop levels or R-loop-associated responses in cancer cells could be potential biomarkers for efficacy of drugs that exacerbate R-loop-dependent DNA damage. A model has been proposed to estimate R-loop levels and their prognostic value in cancer RNA-sequencing datasets (Zhang et al., 2024), and PARP1 activation at R-loops has been suggested as a potential predictive biomarker for sensitivity to PARP and ATR inhibition (Liu et al., 2024). Thus, understanding the mechanisms of RNA:DNA hybrid and R-loop regulation in cancer is imperative to developing new methods that target R-loops or use their presence as biomarkers to direct treatment choice.

## RNase H2 in cancer development and treatment

RNase H2 loss has been implicated in tumorigenesis using tissue-specific knock-out mouse models (Reijns et al., 2012). *RNASEH2B* deletion in epithelial cells causes spontaneous DNA damage and p53-dependent apoptosis. However, bowel tumorigenesis occurs when *RNASEH2B* is concomitantly ablated with *TP53* in intestinal epithelial cells (Aden et al., 2019). Thus, RNASEH2B has been suggested to suppress colorectal tumorigenesis by maintaining genomic integrity (Aden et al., 2019). RNase H2 deficiency also predisposes mice to squamous cell carcinoma, attributed to the loss of RER (Hiller et al., 2018). A rare germline amino acid variant of human RNASEH2C has been correlated with significantly increased risk of breast cancer, potentially by decreasing RNase H2 complex stability (Zhang et al., 2014). Due to its proximity to two tumour suppressor loci, *RNASEH2B* can be collaterally deleted in human cancers. This occurs in 43% of chronic lymphocytic leukaemia cases when DLEU2-mir-15-6 microRNA is deleted, and in 34% of CRPCs associated with *RB1* deletion (Zimmermann et al., 2018). *RNASEH2B* deletion has been exploited for synthetic-lethal approaches, where RNase H2 deficiency sensitises cells to PARP or ATR inhibition (Wang et al., 2019; Zimmermann et al., 2018, 2022). Interestingly, patients with AGS caused by monogenic variants of one of the three RNASEH2 genes are not at increased risk of cancer (Crow et al., 2015), which could be explained by early mortality or by residual RNase H2 activity due to hypomorphic RNase H2 variants being enough to maintain genomic integrity and suppress tumorigenesis.

Whereas RNase H2 loss can contribute to cancer initiation, accumulating evidence suggests that RNase H2 upregulation correlates with progression, metastasis and treatment resistance in solid tumours and haematological malignancies (Table 1).

**Table 1.** Alterations in *RNASEH2A, RNASEH2B* and *RNASEH2C* in cancer

Cancer	Subtype	*RNASEH2A*	*RNASEH2B*	*RNASEH2C*	Reference
Bladder	Urothelial carcinoma	mRNA up	–	–	Marsili et al., 2021
	Carcinoma	–	Deletion	–	Zimmermann et al., 2022
Blood (leukaemia)	Acute myeloid leukaemia	mRNA up	–	–	Flanagan et al., 2009
	B-cell acute lymphoblastic leukaemia	mRNA up	–	–	Flanagan et al., 2009
	Chronic lymphocytic leukaemia	–	Collateral deletion	–	Zimmermann et al., 2018
	T-cell acute lymphoblastic leukaemia	mRNA up	–	–	Flanagan et al., 2009
Bone	Chondrosarcoma	mRNA up	–	–	Flanagan et al., 2009
Brain	Glioblastoma multiforme	mRNA up	–	–	Flanagan et al., 2009; Marsili et al., 2021
	Oligodendroglioma	mRNA up	–	–	Flanagan et al., 2009; Marsili et al., 2021
	Oligoastrocytoma	mRNA up	–	–	Flanagan et al., 2009; Marsili et al., 2021
Breast	ER positive	mRNA up	–	–	Flanagan et al., 2009; Shen et al., 2020
	Triple negative	mRNA up	–	–	Flanagan et al., 2009; Marsili et al., 2021; Yuan et al., 2017
	N/A	–	–	Single amino acid variant increases cancer risk	Zhang et al., 2014
Cervical	N/A	mRNA up	–	–	Xu et al., 2019
	Squamous cell carcinoma	mRNA up	–	–	Marsili et al., 2021
Colorectal	Colon and rectum adenocarcinoma	mRNA up	–	–	Marsili et al., 2021
	N/A	mRNA up	–	–	Zhang et al., 2018
	N/A	mRNA up	mRNA up (metastasis)	mRNA up (metastasis)	Yang et al., 2017
Gastric	Adenocarcinoma	mRNA up	mRNA up	–	Marsili et al., 2021; Mottaghi-Dastjerdi et al., 2015
Germ cell	Seminoma	mRNA up	–	–	Flanagan et al., 2009
Head and neck	Squamous cell carcinoma	mRNA up	–	–	Flanagan et al., 2009; Marsili et al., 2021
Kidney	Renal cell carcinoma	mRNA up	–	–	Flanagan et al., 2009; Yang et al., 2018
Liver	Hepatocellular carcinoma	mRNA up	–	–	Hao et al., 2023; Hua et al., 2020; Zhao et al., 2022
Lung	Adenocarcinoma	mRNA up	–	–	Marsili et al., 2021; Zhang et al., 2021
	Squamous cell carcinoma	mRNA up	–	–	Marsili et al., 2021; Zhang et al., 2021
Neuroendocrine	Pheochromocytoma	mRNA up	–	–	Marsili et al., 2021
	Paraganglioma	mRNA up	–	–	Marsili et al., 2021
Oesophageal	Carcinoma	mRNA up	–	–	Marsili et al., 2021
Pancreas	Carcinoma	mRNA up	–	–	Marsili et al., 2021
Prostate	Castration resistant	mRNA up	–	–	Kimura et al., 2022
		–	Collateral deletion	–	Zimmermann et al., 2018
	N/A	mRNA up	mRNA down	–	Kimura et al., 2022; Williams et al., 2014
		–	Collateral deletion	–	Miao et al., 2022
Skin	Melanoma	mRNA up	–	–	Flanagan et al., 2009
Thyroid	Carcinoma	mRNA up	–	–	Marsili et al., 2021
Uterus	Uterine corpus endometrial carcinoma	mRNA up	–	–	Flanagan et al., 2009; Nakazawa et al., 2024
	Uterine leiomyosarcoma	–	Deletion	–	Nakazawa et al., 2024

N/A, cancer subtypes not specified in the study.

*RNASEH2A* upregulation has been observed independently of *RNASEH2B* or *RNASEH2C* (Flanagan et al., 2009; Kimura et al., 2022). Conversely, upregulation of *RNASEH2B* correlates with upregulation of *RNASEH2C*, but not of *RNASEH2A*, suggesting differential regulation of the three subunit-encoding genes (Marsili et al., 2021; Yang et al., 2017; Zhang et al., 2018). The *RNASEH2A* gene is a target of E2F cell cycle-associated transcription factors (Sugawara et al., 2022), and increased *RNASEH2A* expression has been correlated with proliferative human tissues and with proliferation markers in cancer (Marsili et al., 2021). If RNase H2 subunits are differentially regulated with possibly independent roles, this could change the conclusions of previous studies that depleted, deleted or overexpressed only one RNase H2 subunit. Nevertheless, because RNase H2 subunits are co-regulated at the protein level (Reijns et al., 2011), the protein levels of all subunits could still increase regardless of which subunit mRNA is upregulated (Wilkins et al., 2025).

RNASEH2A is upregulated at the mRNA and protein level in CRPC compared to localised prostate cancer, and high *RNASEH2A* expression correlates with poor prognosis and has been suggested to be a hallmark of progression (Kimura et al., 2022). In human glioma cell lines, *RNASEH2A* upregulation is associated with increased proliferation, and RNASEH2A depletion reduces proliferation and elicits apoptosis (Dai et al., 2016). In lung squamous adenocarcinoma and colorectal adenocarcinoma, increased *RNASEH2A* expression has been linked to cancer progression (Marsili et al., 2021), and *RNASEH2A* is one of few genes found to be upregulated in response to oncogenic transformation of human mesenchymal cells (Flanagan et al., 2009). *RNASEH2A* upregulation has further correlated with chemoresistance in oestrogen receptor (ER)-positive breast cancer (Shen et al., 2020) and has been suggested as a prognostic biomarker in renal cancer (Yang et al., 2018). In hepatocellular carcinoma (HCC), increased *RNASEH2A* expression in response to hypoxia correlates with suppression of cGAS–STING signalling and thus evasion of immunologic stress, and is also associated with poor prognosis and reduced overall survival (Hao et al., 2023; Zhao et al., 2022).

*RNASEH2B* and *RNASEH2C* expression levels are upregulated in metastatic colorectal carcinomas, as compared to paired non-metastatic colorectal carcinomas (Marsili et al., 2021; Yang et al., 2017; Zhang et al., 2018). In ovarian cancer, low expression of *RNASEH2B* or *RNASEH2C,* but not of *RNASEH2A*, correlates with higher progression-free survival, suggesting that expression of these subunits could be a prognostic marker (Polaczek et al., 2020). *RNASEH2C* is a potential metastasis susceptibility gene in breast cancer, independently of RNase H2 enzymatic functions. Ectopic RNASEH2C overexpression increases pulmonary breast cancer metastasis in a mouse orthotopic xenograft model (Deasy et al., 2019). However, in HCC, *RNASEH2A* expression positively correlates with markers of epithelial–mesenchymal transition, an early process in metastasis, raising the question of whether correlation with metastasis is always specific to *RNASEH2B* and *RNASEH2C* (Hao et al., 2023).

## Conclusions

Dysregulation of RNase H2 is associated with cancer at all stages. RNase H2 upregulation could be associated with altered RS and RS responses, including responses to RS-inducing cancer treatments such as CPT (Audoynaud et al., 2023; Goehring et al., 2024; Heuzé et al., 2023). The role of RNase H2 in maintaining genomic integrity can be a barrier to tumour development but might also benefit established cancer cells, promoting their survival and potentially their resistance to therapies. Despite this, the underlying molecular mechanisms of altered RNase H2 in cancer, the importance of the multiple roles that RNase H2 has at replication forks and in TRCs, and the potential of RNase H2 as a therapeutic target remain poorly understood. Outstanding questions include those around the relative contributions and regulation of the dual enzymatic functions of RNase H2, as well as potential activity-independent functions of individual subunits in RS and cancer.

Although correlating alterations in RNase H2 subunit levels to cancer is important, information on cellular RNase H2 activity in cancer is essential (Schulz et al., 2023). Experimentally, RNase H2 activity can be measured by a fluorescence resonance energy transfer-based assay *in vitro* using either recombinant RNase H2 or whole-cell lysates (Crow et al., 2006; Parniak et al., 2003; Reijns et al., 2012; White et al., 2013). This method has recently been adapted and standardised for clinical screening of RNase H2 activity directly in patient whole-cell lysates (Schulz et al., 2023). Such approaches could be important to determine RNase H2 activity in response to cancer therapies.

Due to its roles in resolving RNA:DNA hybrids and preventing RS, targeting of RNase H2 in cancer is being investigated as a viable therapeutic strategy, including in combination with immune checkpoint inhibitors (Box 1). Depletion of RNASEH2A or RNASEH2B reduces the viability of T-cell leukaemia cell lines, due to loss of NHEJ activity and accumulation of DSBs (Ghosh et al., 2022). Compounds that can inhibit RNase H2 *in vitro* and decrease cell viability have been reported (Kim et al., 2013; White et al., 2013; Wang et al., 2025), and treatment with these inhibitors could shrink CRPC tumours in nude mice (Kimura et al., 2022). However, the specificity of these compounds for inhibiting RNase H2 in a cellular context has yet to be confirmed. If specific RNase H2 inhibitors were to be established, this could open substantial new possibilities in probing the roles of RNA:DNA hybrids and RNase H2 in RS, GIN, cancer development and cancer treatment.

Box 1. Targeting RNase H2 in cancer Loss of RNase H2 can cause spontaneous DNA damage and RS by increasing the frequency of genome-embedded rNMPs and RNA:DNA hybrids (Aden et al., 2019; Cristini et al., 2022; Hiller et al., 2018; Pizzi et al., 2015). RNase H2 is upregulated in cancer, suggesting a reliance on RNase H2 to maintain genomic integrity for survival. This provides a strategy for targeting RNase H2 activity with small-molecule inhibitors, such as R11 and R14 (White et al., 2013) (see box figure). Pharmacological RNase H2 inhibition can shrink CRPC tumours in nude mice (Kimura et al., 2022). A recent pharmacokinetic and metabolic evaluation of R14 shows that there is interest in progressing RNase H2 inhibitors for pre-clinical drug development (Wang et al., 2025).

*RNASEH2B* collateral deletions in cancer have been exploited as a genetic vulnerability for synthetic lethal treatment with PARP inhibitors (PARPi) or ATR inhibitors (ATRi) (Carmichael et al., 2024; Miao et al., 2022; Wang et al., 2019; Zimmermann et al., 2018, 2022). Recent work suggests that RNase H2 subunits and cellular activity (Wilkins et al., 2025), as well as RNase H2 localisation to HO-TRCs (Goehring et al., 2024), might be responsive to RS produced by the chemotherapeutic agents CPT and HU, and RNASEH2A overexpression in prostate cancer cells can attenuate CPT-induced apoptosis (Kimura et al., 2022). Combining RNase H2 inhibitors with DNA damage response (DDR) inhibitors or chemotherapeutic agents in cancers without RNASEH2 deletion might prove a viable strategy to circumvent RNase H2-mediated RS tolerance, exacerbating DNA damage and cell death.

R-loops and RS can promote cytosolic nucleic acid accumulation, which activates inflammatory responses, such as cGAS–STING signalling (reviewed in Lee et al., 2025). R-loop accumulation can thereby increase anti-tumour immunity and enhance immune checkpoint blockade (ICB) responses, which RNASEH2B overexpression can attenuate (Maxwell et al., 2024). Therefore, RNase H2 inhibition could enhance anti-tumour immunity in a similar manner, potentially synergising with ICB treatment (Anh Nguyen et al., 2025).

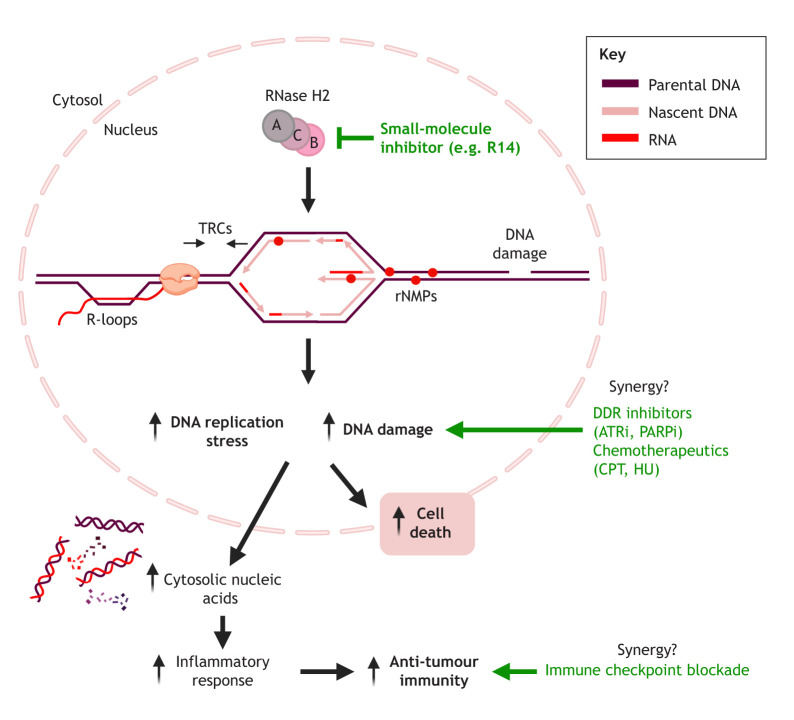

Created in BioRender by Wilkins, R. (2025). https://BioRender.com/oyg653z. This figure was sublicensed under CC-BY 4.0 terms.
